# The Impact of COVID-19-Related Work Stress on the Mental Health of Primary Healthcare Workers: The Mediating Effects of Social Support and Resilience

**DOI:** 10.3389/fpsyg.2021.800183

**Published:** 2022-01-21

**Authors:** Lu-shao-bo Shi, Richard Huan Xu, Yi Xia, Dong-xue Chen, Dong Wang

**Affiliations:** ^1^School of Health Management, Southern Medical University, Guangzhou, China; ^2^Department of Rehabilitation Sciences, The Hong Kong Polytechnic University, Hong Kong, Hong Kong SAR, China; ^3^Jockey Club School of Public Health and Primary Care, The Chinese University of Hong Kong, Hong Kong, Hong Kong SAR, China; ^4^Institute of Health Management, Southern Medical University, Guangzhou, China

**Keywords:** COVID-19, primary healthcare workers, work stress, social support, resilience, mental health

## Abstract

**Objective:**

The psychological condition of healthcare workers since the COVID-19 pandemic has attracted the attention of many studies. However, few have reported on psychosocial problems of primary healthcare workers in the COVID-19 pandemic. This study aimed to examine the mediating roles of social support and resilience in COVID-19-related work stress and symptoms of anxiety and depression.

**Methods:**

A total of 840 primary healthcare workers in 17 community health centers in Guangzhou, China, were recruited from May to July 2021. Data on demographic characteristics, COVID-19-related work stress, social support, resilience, anxiety and depression were collected. A structural equation model was used for mediation analysis.

**Results:**

More than half of participants reported mild or more severe (at least borderline abnormal) symptoms of anxiety (68.1%) and depression (55.6%). Social support and resilience mediate the association between COVID-19-related work stress and symptoms of anxiety and depression, respectively. Furthermore, the association between work stress and symptoms of anxiety and depression was also mediated by an accumulation of social support and resilience. The indirect effect of COVID-19-related work stress on anxiety and depression through resilience was much greater than other indirect effects.

**Conclusion:**

Anxiety and depression were prevalent among primary healthcare workers. This study highlights the psychological impact of the COVID-19-related psychosocial work environment on primary healthcare workers. There is an urgent need to improve working conditions for primary healthcare workers in the COVID-19 and to implement intervention strategies aimed at increasing individual resilience alongside the establishment of external supportive work environments.

## Introduction

COVID-19 is still raging in much of the world, posing a huge challenge for populations and societies worldwide to manage their health (Pfefferbaum and North, 2020; Robbins et al., 2021). As of 15 December 2021, a global total of 270,791,973 confirmed cases and 5,318,216 deaths have been reported (World Health Organization (WHO), 2021). After adopting a series of strict and decisive public health measures, COVID-19 prevention and control has been significantly effective in China. However, given that the virus is constantly mutating and there is a risk of infection from outside the country, scattered infection cases are inevitable. To prevent the resurgence of COVID-19, some long-term public health measures are necessary in China, including mass vaccination, the establishment of fever clinics, expansion of nucleic acid testing, and constant supervision of infection prevention and control in medical institutions (Liang et al., 2021). Healthcare systems and labor forces will certainly continue to experience a tremendous burden for a long time due to the constant struggle with the potential risk of infection (Ofei-Dodoo et al., 2021).

Compared with the general population, medical staff is more likely to be exposed to multiple risk factors related to mental health problems, such as discordant doctor-patient relationships, accumulated frustration in the face of patient death, and increased government supervision of professional activities (Paiva et al., 2018; Huo et al., 2021). Furthermore, COVID-19 exposes healthcare workers to an additional psychological burden, including fear of infection, a sense of social isolation, and urgency at work. Previous studies have suggested that healthcare workers experience significant mental burdens and psychological disorders in the COVID-19 pandemic (Lai et al., 2020; Zhang et al., 2020). Reducing damage to the mental health of medical staff caused by COVID-19 is one major challenge of the pandemic (Feng and Yin, 2021). However, research to date has primarily focused on assessing the psychological responses of the entire medical staff (Wang et al., 2020; Xu et al., 2021), with limited attention to primary healthcare workers.

As the gatekeeper of the health system, in addition to providing basic health services, the primary healthcare system is responsible for multiple COVID-19 front-line tasks (e.g., nucleic acid testing, disinfection of public environments, vaccination, and promotion of epidemic prevention knowledge). Notably, with the continuous stacking of epidemic prevention and control policies in China, primary healthcare workers have to take increasing responsibility, such as surveillance and report of patients with fever, technical training on epidemic prevention and control, and health management of discharged COVID-19 patients (e.g., isolation management, return visit and re-examination, health monitoring, rehabilitation medical treatment) (General Office of National Health Commission of the People’s Republic of China, 2020; State Council of the People’s Republic of China, 2020; National Health Commission of the People’s Republic of China, 2021). The current containment measures will be maintained until the global COVID-19 pandemic is declared over. The sustaining work requirements of epidemic prevention and control may negatively impact the daily life, social cognition, and psychological needs of primary healthcare workers, which consequently leads to adverse psychological symptoms. Hence, it is urgent to explore the impact of the psychosocial work environment on the psychological well-being of primary healthcare workers during the COVID-19 pandemic.

According to the effort-reward imbalance (ERI) model, the stress in the work environment involves the following three factors: work effort, work reward, and overcommitment (Siegrist et al., 2004). Specifically, work stress results from excessive work-related commitment and an imbalance between work effort and work reward (e.g., salary, respect, job security, job development prospects, etc.) (Siegrist et al., 2009). For primary healthcare workers, their working situation is associated with the development of COVID-19. In other words, in the context of COVID-19, the work stress situation of primary healthcare workers may have an unforeseen impact. Indeed, considerable evidence suggests that work stress is closely related to negative mental health outcomes (Reichenberg and MacCabe, 2007; Kopp et al., 2008), and prolonged, high levels of work stress directly contribute to anxiety and depressive disorders (Weinberg and Creed, 2000; Magnavita et al., 2021). However, the internal factors and underlying mechanisms of this relationship in the context of COVID-19 remain unclear. Therefore, given the impact of work stress and its negative effect on mental health, it is essential to explore the process and mediating factors of the transformation of COVID-19-related work stress into anxiety and depression in primary healthcare workers. Based on the above evidence, hypothesis 1 was proposed: COVID-19-related work stress positively predicts anxiety and depression among primary healthcare workers (H1).

Social support is defined as an individual’s access through social ties to other individuals, groups, and the larger community, which is a social interaction process related to altruism, sense of obligation, and reciprocity (Lin et al., 1979; Hofman et al., 2021). According to coping theory, social support is one of the main coping strategies of individuals facing stress, and reduces the possible negative effects of stressful events by solving problems (i.e., problems are solved by getting information and practical help from social ties) and easing emotions (i.e., regulating negative emotional responses through social ties) (Lazarus, 1993; Mo et al., 2020). The beneficial impacts of social support on health and well-being have been widely recognized. Specifically, previous studies have confirmed that social support not only directly brings well-being, but also promotes mental health by buffering the adverse effects of stressors (Cohen and Wills, 1985; Sun et al., 2020). Several studies have found that social support is an important source of positive psychological qualities (e.g., self-efficacy) (Bhattarai et al., 2021). Moreover, social support protects against psychological problems (e.g., anxiety, depression, PTSD, suicidal ideation) (Dour et al., 2014; Arenson et al., 2021; Zalta et al., 2021). Overall, social support is a key protective factor for mental health and has the potential to improve stress coping and social adaptability (Zhang X. et al., 2021). Therefore, based on the above evidence, hypothesis 2 was proposed: social support mediates the association between COVID-19-related work stress and symptoms of anxiety and depression in primary healthcare workers (H2).

Resilience is a personal quality that enables individuals to recover and flourish following stressful events. Resilience refers to the dynamic adaptive process of adversity, trauma, tragedy, threats, or significant stressors (Bonanno, 2004; Southwick et al., 2014). Resilience has been recognized as an internal factor that is closely related to positive emotional characteristics, which mobilize positive emotions (e.g., humor, optimism) to cope with stressful events (Tugade et al., 2004). In general, resilience can improve psychological well-being by encouraging better coping strategies (Thompson et al., 2018). Hence, resilience may mediate the process of negative mental health outcomes (e.g., anxiety, depression) triggered by stressful events (Kumpfer, 2002; Zhang D. et al., 2021). Notably, although the robust relationship between COVID-19-related stress, resilience, anxiety, and depression has been consistently shown among healthcare workers (Mosheva et al., 2020), the resilience mechanism underlying this relationship has not been elucidated. Therefore, based on the above evidence, hypothesis 3 was proposed: resilience mediates the association between COVID-19-related work stress and symptoms of anxiety and depression in primary healthcare workers (H3).

In this study, social support and resilience were considered as external and internal factors, respectively, that mediate the association between COVID-19-related work stress and the symptoms of anxiety and depression. Indeed, the relationship between COVID-19-related work stress and mental health is also likely to be influenced by the combination of social support and resilience. On the one hand, the close association between social support and resilience has been unanimously agreed upon in the existing literature. Stable and diversified social ties can provide external support resources for individuals to adapt to adversity, and subsequently produce positive results (Kumpfer, 2002; Bhattarai et al., 2021). On the other hand, extensive evidence suggests that social support is an important source of resilience for healthcare workers (Park et al., 2020; Wu C. et al., 2021), and resilience mediates the association between social support and adverse mental health outcomes (e.g., Depression) (Li et al., 2015). Therefore, based on the above evidence, hypothesis 4 was proposed: COVID-19-related work stress affected anxiety and depression of primary healthcare workers through the sequential mediating effects of social support and resilience (H4).

**FIGURE 1:**
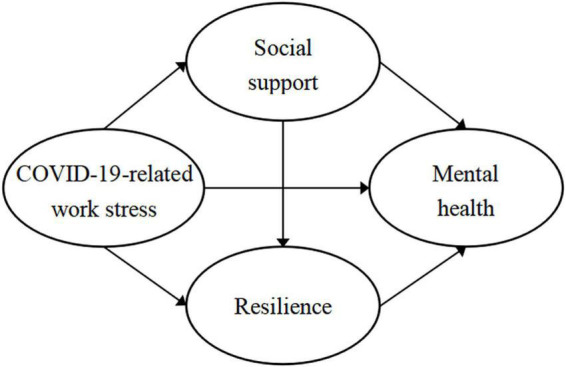
Multiple mediating hypothesis model between variables. Hl, COVID-19-related work stress→Mental health; H2, COVID-19-related work stress→Social support→Mental health; H3, COVID-19-related work stress→Resilience→Mental health; H4, COVID-19-related work stress→Social support→Resilience→Mental health.

Therefore, to reveal the complex relationship between COVID-19-related work stress and mental health, an integrated multiple mediating model was adopted in this study (Figure 1). This study aimed to understand the mental health level of primary healthcare workers by measuring anxiety and depression symptoms, and to investigate the mediating roles of social support and resilience in the relationship between COVID-19-related work stress and symptoms of anxiety and depression. This study thus provides a scientific basis for preventing psychological problems and formulating relevant intervention measures for primary healthcare workers in the COVID-19 pandemic.

## Materials and Methods

### Participants and Data Collection

A cross-sectional field survey on primary healthcare workers in community health centers was conducted from May to July 2021 in Guangzhou City, Guangdong Province, China. Given the differences in economic development level and street division among different districts, 17 community health centers from six districts in the central city of Guangzhou were selected by stratified random sampling. Specifically, using each district as a sample layer, a minimum of 15% of community health centers in each district was decided to be included. Overall, there are 19, 18, 18, 26, 18, and 13 community health centers in Liwan, Yuexiu, Haizhu, Tianhe, Baiyun, and Huangpu, respectively. According to our sampling method, a certain number of community health centers were selected in each district (Haizhu, 2; Huangpu, 2; Liwan, 3; Yuexiu, 3; Tianhe, 3; and Baiyun, 4). All primary healthcare workers who met the following inclusion criteria were recruited at the 17 community health centers: (1) volunteered to participate in the questionnaire survey after providing informed consent; (2) were regular employees; and (3) had been working in the center for the last 21 months.

This study was approved by the Ethics Committee of Southern Medical University, Guangzhou, Guangdong Province, China (Ethical approval number: NFYKDX002). Before the survey, each participant was informed of the purpose of the study and it was emphasized that their answers were voluntary, anonymous, and confidential. With the support of selected community health centers, we distributed questionnaires to primary medical staff. Under the guidance of researchers, participants filled out the questionnaire by themselves, which took an average of 6 min to complete. In this study, a total of 1020 primary healthcare workers were recruited to participate in the survey; 840 questionnaires were eventually included in the statistical analysis, with an effective response rate of 82.4%.

The following considerations were taken to ensure the reasonableness and rigor of the study: First, to avoid excessive collinearity among variables due to the use of similar items in different questionnaires, there were no overlapping factors measured by the assessment tools. Second, during the development process of the questionnaire of COVID-19-related work stress, several experts from the psychology, management, and statistics community were invited to make modifications to the content and structure of the questionnaire to ensure its reliability and validity. Third, all items in the questionnaire were self-reported in Chinese and conformed to Chinese cultural characteristics. Finally, researchers received unified training on the links between paper questionnaire issuance and data building to reduce the impact of researchers’ subjective bias on the data authenticity.

### Measures

#### Demographic Characteristics

Participants’ basic information, such as sex, age, educational level, marital status, working years, occupation, and personal monthly income were collected.

#### COVID-19-Related Work Stress

In this study, COVID-19-related work stress was defined as the difference in perceived levels of work stress before and after the pandemic. To assess COVID-19-related work stress, a 15-item questionnaire was constructed based on the ERI model (Siegrist et al., 2004). The questionnaire was composed of three dimensions, as follows: effort (E, 3 items); reward (R, 8 items); and overcommitment (OC, 4 items). The items were designed to examine the difference in perceived levels of work stress before and after the pandemic. An example of the questionnaire items is as follows: Compared to before the COVID-19, I get more easily overwhelmed by time pressures at work. In the ERI model, effort and overcommitment are the external environments and individual cognitive independent factors of work-related performance, respectively (Siegrist et al., 2009). Previous studies have validated the measurement structure of the ERI questionnaire in China (Li et al., 2012). All items were scored on a Likert 4-point scale ranging from 1 to 4. To eliminate the different numbers of items difference between the two dimensions, the effort-reward ratio was obtained using the _(E/3)/(R/8)_ correction formula, which reflects the imbalance between the effort and reward of COVID-19-related work. E is the total score of the effort dimension and R is the total score of the reward dimension. When the effort-reward ratio was greater than 1, participants were considered to be in a state of high effort and low reward. A higher effort-reward ratio and overcommitment score indicated a higher level of work stress. In the present study, the standardized Cronbach’s Alpha of the questionnaire of COVID-19-related work stress was 0.849. In addition, the psychometric characteristics of the self-designed questionnaire of COVID-19-related work stress have been comprehensively validated (Supplementary Table 1).

#### Social Support

The Social Support Rating Scale (SSRS) was used to measure the level of social support and includes 10 self-reported items in Chinese (Xiao, 1994). It consists of the three following subscales: objective support (OS, 3 items), subjective support (SS, 4 items), and use of support (UOS, 3 items). The total Social Support Rating Scale score ranges from 12 to 66 and is obtained by calculating the sum of the three subscale scores. Higher scores indicate a higher level of social support. The Social Support Rating Scale is regarded as one of the most suitable tools for assessing social support in the Chinese population, and an excellent reliability and validity have been demonstrated in different surveys (Yu et al., 2020; Zhu et al., 2021). In the present study, the standardized Cronbach’s Alpha of SSRS was 0.770.

#### Resilience

The Brief Resilience Scale was used to evaluate resilience. The scale was divided into two parts, as follows: a positive polarity factor, measured using 3 positively worded items (forward coding); and a negative polarity factor that was measured using 3 negatively worded items (reverse coding) (Smith et al., 2008; Fung, 2020). Each item is scored on a 5-point Likert scale ranging from 1 to 5. As a reliable tool for measuring individual elasticity, the internal consistency, convergence validity, and structural validity of the Brief Resilience Scale in the Chinese population have been verified (Windle et al., 2011; Fung, 2020). In the present study, the standardized Cronbach’s Alpha of the brief resilience scale was 0.850.

#### Anxiety and Depression

The Hospital Anxiety and Depression Scale (HADS) was used to assess anxiety and depression levels (Zigmond and Snaith, 1983). In this scale, 7 items are assigned to measure anxiety (HADS-A, 1 item scored in reverse) and the other 7 items are used to measure depression (HADS-D, 5 items scored in reverse). Each item was scored from 0 to 3, and the total score for anxiety or depression ranged from 0 to 21. In each subscale, scores ranging from 0 to 7, 8 to 10, and 11 to 21 were interpreted as normal, borderline abnormal, and abnormal, respectively (Tasnim et al., 2021). The HADS has been widely used to assess mental health in different groups of people, including medical staff, owing to its excellent psychometric characteristics (Rahman et al., 2019; Khanal et al., 2020). In the present study, the standardized Cronbach’s Alpha of the HADS-A and HADS-D was 0.882, 0.822, respectively.

### Statistical Analysis

Descriptive analysis was performed on all variables, including demographic characteristics. Continuous variables and categorical variables are presented as the mean (standard deviation) and frequency (percentage), respectively. One-sample Kolmogorov–Smirnov Test was applied to confirm whether the variables conform to normal distribution. Spearman’s Rank Correlation was used to assess the correlations between measures. A correlation coefficient less than 0.3 indicates a mild correlation effect. A structural equation model was used to verify the study model. Absolute fit indices (goodness of fit index, standardized root mean squared residual, and root mean square error of approximation) and incremental fit indices (comparative fit index, Tucker–Lewis index, and normed fit index) were calculated using the maximum likelihood estimation to examine the model fit. Specifically, when the goodness of fit index, comparative fit index, Tucker–Lewis index, and normed fit index values were >0.9, and the standardized root mean squared residual and root mean square error of approximation were <0.08, the hypothetical model was broadly perceived as a good fit (Hu and Bentler, 1999; Kenny and McCoach, 2003; Groarke et al., 2021). Bootstrap tests with 5,000 random samples and 95% confidence intervals (CIs) were used to analyze the significance of the mediating role. Descriptive analysis and Cronbach’s alpha test were conducted using SPSS v25.0, and confirmatory factor analysis, structural equation model, and bootstrap tests were conducted using AMOS v25.0.

## Results

### Common Method Bias Testing

Common method bias is likely to result in systematic errors in the verification of mediation relationships. The potential impact of common method bias was measured by The Harman single-factor test before data analysis (Podsakoff et al., 2003). Eight factors with eigenvalues greater than 1 were obtained after exploratory factor analysis without rotation setup, and the first factor explains 24.6% of the total variance, which was less than 40% of the critical criterion, indicating that the influence of common method bias on the results of the statistical analysis in this study was absence of serious.

### Participants’ Sociodemographic Characteristics and Incidence of Anxiety and Depression

A total of 840 primary healthcare workers participated in this study. As shown in Table 1, 174 (20.7%) were male, and 666 (79.3%) were female. The mean age of the participants was 36.8 years (SD = 8.70). The majority of respondents had a bachelor’s degree or above (75.4%) and were married (76.7%). The average number of working years of the participants was 8.85 years (SD = 7.83). The percentages of physicians, nurses, medical technicians, and management support personnel were 43.8, 37.9, 14.8, and 3.5%, respectively. More than half of the respondents had monthly incomes ranging from 3,000 to 9,000 RMB (64.2%). Of the 840 participants, 209 (24.9%) reported mild symptoms of anxiety, and 363 (43.2%) were identified as having severe anxiety symptoms. Similarly, 305 (36.3%) participants had mild depression symptoms, and 162 (19.3%) were identified as having severe depressive symptoms.

**TABLE 1:** Sociodemographic characteristics of primary healthcare workers.

Variables	Frequency (*N*)	Percentage(%)
**Sex**		
Male	174	20.7
Female	666	79.3
**Age**		
20–29	174	20.7
30–39	385	45.8
40–49	182	21.7
50 or above	99	11.8
**Educational level**		
Technical secondary school and below	34	4.0
Junior college	173	20.6
Bachelor	606	72.2
Master degree or above	27	3.2
**Marital status**		
Single	169	20.1
Married	644	76.7
Divorced/Widowed	27	3.2
**Working years**		
1–5	343	40.8
6–10	279	33.2
11 or above	218	26.0
**Occupation**		
Physician	368	43.8
Nurse	318	37.9
Medical technician	125	14.8
Management support personnel	29	3.5
**Personal monthly income (RMB)**		
3000 or below	45	5.4
3000–6000	299	35.6
6000–9000	240	28.6
9000–12000	156	18.5
12000–15000	58	6.9
15000 or above	42	5.0
**Anxiety**		
Normal (HADS-A≤7)	268	31.9
Borderline abnormal (8≤HADS-A≤10)	209	24.9
Abnormal (11≤HADS-A≤21)	363	43.2
**Depression**		
Normal (HADS-D≤7)	373	44.4
Borderline abnormal (8≤HADS-D≤10)	305	36.3
Abnormal (11≤HADS-D≤21)	162	19.3

*An average exchange rate of RMB against USD was 6.4439.*

### Descriptive Statistics and Correlations Between Key Variables

The mean (M) and standard deviation (SD) of, and correlation coefficients between the measures are displayed in Table 2. Given the results of the One-sample Kolmogorov-Smirnov Test showed the hypothesis of the normal distribution is not supported (Supplementary Table 2), the application of Spearman’s Rank Correlation was accepted. The effort-reward ratio and overcommitment were positively correlated with anxiety and depression, and negatively correlated with social support and resilience (*P* < 0.05). Furthermore, social support, objective support, subjective support, use of support, positive polarity factor, negative polarity factor, and resilience were negatively correlated with anxiety and depression (*P* < 0.01).

**TABLE 2:** Descriptive statistics and bivariate Spearman’s rank correlation among study variables.

	(1)	(2)	(3)	(4)	(5)	(6)	(7)	(8)	(9)	(10)	(11)	M (SD)
(1) E-R ratio	1											1.27 (0.44)
(2) OC	0.457**	1										11.34 (2.49)
(3) Social support	−0.210**	−0.075*	1									40.80 (8.48)
(4) OS	−0.149**	−0.075*	0.808**	1								10.41 (3.68)
(5) SS	−0.212**	−0.066	0.882**	0.504**	1							22.51 (4.99)
(6) UOS	−0.135**	−0.055	0.548**	0.372**	0.335**	1						7.88 (1.70)
(7) Resilience	−0.346**	−0.343**	0.359**	0.290**	0.319**	0.218**	1					3.19(0.61)
(8) PPF	−0.312**	−0.233**	0.322**	0.255**	0.286**	0.204**	0.845**	1				3.27 (0.62)
(9) NPF	−0.310**	−0.361**	0.314**	0.255**	0.279**	0.200**	0.893**	0.542**	1			3.11 (0.73)
(10) Anxitey	0.310**	0.458**	−0.305**	−0.232**	−0.286**	−0.185**	−0.594**	−0.498**	−0.539**	1		9.68 (4.31)
(11) Depression	0.314**	0.367**	−0.392**	−0.327**	−0.329**	−0.263**	−0.559**	−0.473**	−0.507**	0.705**	1	7.64 (3.78)

*P<0.05; **P<0.01; M, mean, SD, standard deviation; E-R ratio, Effort-Reward ratio; OC, overcommitment; OS, objective support; SS, subjective support; UOS, use of support; PPF, positive polarity factor; NPF, negative polarity factor.

### Measurement Model

The measurement model consisted of four constructs – COVID-19-related Work Stress, social support, resilience, and mental health. The analysis results for the measurement model are presented in Table 3. All factor loadings of the measurement model were significant (*P* < 0.001), and the standardized factor loading ranged from 0.534 to 0.863. Moreover, the measurement model revealed a good fit with the data (χ2/df = 3.740, *P* < 0.001, goodness of fit index = 0.980, comparative fit index = 0.979, Tucker–Lewis index = 0.962, normed fit index = 0.971, standardized root mean squared residual = 0.034, root mean square error of approximation = 0.057).

**TABLE 3:** Maximum likelihood parameter estimates for measurement model.

Path	Factor loadings	S.E.	Standardized factor loadings	*P*
E-R ratio<—COVID-19-related work stress	1		0.611	
OC<—COVID-19-related work stress	6.923	0.568	0.739	<0.001
OS<—Social support	1		0.703	
SS<—Social support	1.422	0.102	0.737	<0.001
UOS<—Social support	0.351	0.029	0.534	<0.001
PPF<—Resilience	1		0.761	
NPF<—Resilience	1.218	0.064	0.788	<0.001
Anxitey<–Mental health	1		0.863	
Depression<—Mental health	0.856	0.033	0.843	<0.001

*E-R ratio, Effort-Reward ratio; OC, overcommitment; OS, objective support; SS, subjective support; UOS, use of support; SE, standard error; PPF, positive polarity factor; NPF, negative polarity factor.*

### Structural Model and Bootstrap Test

**FIGURE 2:**
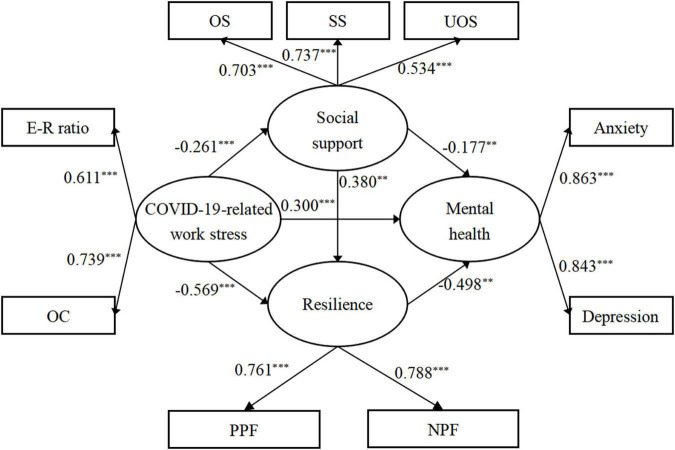
Multiplemediation models with significantly standardized estimates. **P < 0.05; ***P < 0.001, E-R ratio, Effort-Reward ratio; OC, overcommitment; OS, objective support; SS, subjective support; UOS, use of support; PPF, positive polarity factor; NPF, negative polarity factor.

As predicted, all paths in the study model were significant (Figure 2). COVID-19-related work stress (β = 0.300, *P* < 0.001, 95% CI = 0.149 to 0.438) had a significant positive effect on anxiety and depression, while social support (β = −0.177, *P* = 0.001, 95% CI = −0.263 to −0.089) and resilience (β = −0.498, *P* = 0.001, 95% CI = −0.653 to −0.334) had a significant negative association with anxiety and depression. COVID-19-related work stress had a significant negative influence on social support (β = −0.261, *P* < 0.001, 95% CI = −0.378 to −0.144) and resilience (β = −0.569, *P* < 0.001, 95% CI = −0.675 to −0.463). Social support played a significant positive predictive role on resilience (β = 0.380, *P* = 0.001, 95% CI = 0.276 to 0.473).

The 95% CI of the model path was obtained using the bootstrap method. In this process, repeated sampling was performed 5000 times. As outlined in Table 4, the CI of each path coefficient did not contain 0, indicating that the indirect and direct effects were statistically significant. Among the three mediating paths, COVID-19-related work stress → resilience → mental health had the greatest value of indirect effect (β = 0.283, *P* < 0.001, 95% CI = 0.190 to 0.414), followed by COVID-19-related work stress → social support → resilience → mental health (β = 0.049, *P* < 0.001, 95% CI = 0.028 to 0.081) and COVID-19-related work stress→ social support → mental health (β = 0.046, *P* < 0.001, 95% CI = 0.022 to 0.083). Overall, indirect and direct effects accounted for 55.8% and 44.2% of the total effect, respectively, and this model explained 68.9% of the total variance of anxiety and depression.

**TABLE 4:** Standardization direct effects and indirect effects in the model.

	Standardized estimate	*P*	95% confidence interval		Ratio of effect
			Lower	Upper	
Indirect effects	0.378	<0.001	0.275	0.523	55.8%
COVID-19-related work stress→Social support→Resilience→Mental health	0.049	<0.001	0.028	0.081	7.2%
COVID-19-related work stress→Social support→Mental health	0.046	<0.001	0.022	0.083	6.9%
COVID-19-related work stress→Resilience→Mental health	0.283	<0.001	0.190	0.414	41.7%
Direct effects	0.300	<0.001	0.149	0.438	44.2%
Total effects	0.678	<0.001	0.604	0.753	

## Discussion

Currently, there are limited reports on the prevalence of anxiety and depression in primary healthcare workers during the COVID-19 pandemic. The present study revealed the prevalence of anxiety (68.1%) and depression (55.6%) among the participants, which were much higher than the pooled prevalence in overall healthcare workers reported in several meta-analyses in the COVID-19 pandemic (Hao et al., 2021; Pappa et al., 2021; Saragih et al., 2021; Wu T. et al., 2021). Furthermore, we examined the direct and indirect effects of COVID-19-related work stress on anxiety and depression in primary healthcare workers. Social support and resilience were found to independently and continuously mediate the effects of COVID-19-related work stress on anxiety and depression, with a total indirect effect of 55.8%.

Our findings suggested that COVID-19-related work stress is an important predictor of anxiety and depression symptoms among primary healthcare workers, which is consistent with previous studies on occupational health among medical staff. For example, Gao et al. showed that work content, ERI, and overcommitment were significantly associated with anxiety symptoms in nurses (Gao et al., 2012). Bernburg et al. (2016) reported significant associations between working conditions and depressive symptoms among physicians. Indeed, since the COVID-19 outbreak, primary healthcare workers have been exposed to oppressive work environments that create stable stressors (e.g., COVID-19-related work insecurity, overload, responsibility, programmatic work content, and rigor requirements). The long-term accumulation of these stressors may trigger a range of COVID-19-related psychosocial responses, further amplifying the psychiatric hazards of COVID-19 outbreaks for primary health care workers. Therefore, future interventions should focus on the impact of working conditions on primary healthcare workers and provide them with adequate work benefits, solid work security, and ongoing mental health services to support their coping strategies (Ashley et al., 2021).

Our study confirmed the mediating role of social support in the association between COVID-19-related work stress and symptoms of anxiety and depression among primary healthcare workers. This finding is consistent with that of some previous studies conducted with nurses (Wu et al., 2011; Chen et al., 2020). Healthcare workers with high levels of social support may have more chances to adopt a positive coping style, which can reduce anxiety and depression symptoms during the COVID-19 pandemic (Zhu et al., 2020). However, unlike previous studies, we found that social support mediated only 6.9% of the effect of COVID-19-related work stress on anxiety and depression. A possible explanation is that the work restrictions of social distancing, lockdown, and quarantine prevent primary healthcare workers from effectively utilizing their support systems as they did previously, thus weakening the stress-buffering effect of social support (Szkody et al., 2020). Therefore, the beneficial effects of social support cannot be ignored. The primary healthcare institutions must provide a supportive work environment (e.g., using online social networking platforms, limiting the shift time, setting up special rest areas, and providing accurate information about the virus) on the premise of the virus protection requirements, strengthening the social and emotional connection and active coping strategies of primary healthcare workers in the workplace (Labrague, 2021).

We also found that resilience played a significant mediating role in the association between COVID-19-related work stress and symptoms of anxiety as well as depression among primary healthcare workers, which is in line with previous research (Labrague, 2021). Interestingly, The pathway in which COVID-19-related work stress impacted anxiety and depression through resilience had the greatest impact (41.7%) on all indirect pathways. Highly resilient healthcare workers may have adequate coping resources and positive emotions, can effectively address COVID-19-related stressors and withstand the pandemic-related psychological burden, thus reducing the occurrence of psychological distress (e.g., anxiety, depression, insomnia, and fatigue) (Huffman et al., 2021; Yoruk and Guler, 2021). Given the important role of resilience in mitigating the mental health hazards associated with COVID-19-related work stress, there is a need to deliver interventions that focus on enhancing resilience. For example, interventions such as stress management and resilience training programs (Magtibay et al., 2017), mindfulness-based stress reduction, and cognitive restructuring strategies (Huffman et al., 2021) have been recommended to improve the resilience of primary healthcare workers.

Our study indicated that COVID-19-related work stress affected anxiety and depression in primary healthcare workers through the sequential mediating effect of social support and resilience, which is consistent with Kumpfer’s resilience framework. Successful adaptation in adversity comes from the interaction between an individual’s internal characteristics (e.g., resilience) and the external environment (e.g., social support) (Kumpfer, 2002; Bhattarai et al., 2021). Our findings highlight the unique role of social support and resilience in the processes of mitigating mental health damage from COVID-19-related work stress. Specifically, higher levels of social support can provide more external resources to help change stress perceptions and reassess COVID-19-related work stress as manageable, thus improving individuals’ resilience and reducing the occurrence of adverse mental health symptoms (Tam et al., 2021). Therefore, interventions that focus on both social support and resilience may be more effective in improving coping strategies and reducing the risk of anxiety and depression.

The present study has the following limitations. First, the conclusions obtained are based on cross-sectional data, and causal relationships between variables cannot be determined. Future studies should collect follow-up data at multiple time points to assess the longitudinal variation of the association between these factors at different stages of the COVID-19 pandemic. Second, given the self-report questionnaire-centered assessment method adopted in this study, the data obtained may have recall bias. Third, although a rigorous random sampling method was employed to recruit participants from 17 community health centers, the repeatability needs to be noted, as the sample was only from one city in China. Future studies need to perform random sampling in multiple cities to improve the generalizability of our findings.

Despite these limitations, our study has some novel strengths. First, to our knowledge, our study reports a high prevalence of anxiety and depression symptoms among primary healthcare workers for the first time, which emphasizes the urgency for greater attention to the psychological responses of primary healthcare workers during the COVID-19 pandemic. Second, the current study developed a questionnaire of COVID-19-related work stress based on the ERI model, which provides a new perspective for measuring the impact of work stress caused by the pandemic. Third, we verified that social support and resilience mediate the relationship between COVID-19-related work stress and symptoms of anxiety and depression, which provides evidence for the establishment of mental health-protective mechanisms of social support and resilience in the COVID-19 pandemic.

## Conclusion

Overall, more than half of primary healthcare workers suffered from mild or more severe (at least borderline abnormal) symptoms of anxiety (68.1%) and depression (55.6%). More importantly, this study found that COVID-19-related work stress significantly predicted anxiety and depression. The independent and cumulative mediating effects of social support and resilience on the association between COVID-19-related work stress and symptoms of anxiety and depression were verified by applying a structural equation model. Specifically, COVID-19-related work stress not only affected anxiety and depression independently *via* social support and resilience, but also affected anxiety and depression through the sequential mediating role of social support and resilience. Notably, the indirect effect of COVID-19-related work stress on anxiety and depression through resilience was much higher than that of the other indirect effects in our study. These findings have positive implications for the intervention of mental problems among primary healthcare workers, as well as for the improvement of mental health well-being during COVID-19.

## Data Availability Statement

The raw data supporting the conclusions of this article will be made available by the authors, without undue reservation.

## Ethics Statement

The studies involving human participants were reviewed and approved by the Ethics Committee of Southern Medical University, Guangzhou, Guangdong Province, China (Ethical approval number: NFYKDX002). The patients/participants provided their written informed consent to participate in this study.

## Author Contributions

L-S-BS, RX, and DW contributed to the design of this study. L-S-BS, D-XC, YX, and DW collected the data. L-S-BS, RX, and YX conducted the data analysis. L-S-BS wrote the manuscript. All authors edited, approved, and submitted the final manuscript.

## Conflict of Interest

The authors declare that the research was conducted in the absence of any commercial or financial relationships that could be construed as a potential conflict of interest.

## Publisher’s Note

All claims expressed in this article are solely those of the authors and do not necessarily represent those of their affiliated organizations, or those of the publisher, the editors and the reviewers. Any product that may be evaluated in this article, or claim that may be made by its manufacturer, is not guaranteed or endorsed by the publisher.
