# Supplementary material for: The Impact of COVID-19-Related Work Stress on the Mental Health of Primary Healthcare Workers: The Mediating Effects of Social Support and Resilience
Source: Front Psychol. 2022 Jan 21;12:800183. doi: 10.3389/fpsyg.2021.800183 (PMC8814425; doi:10.3389/fpsyg.2021.800183)
Supplement: Supplementary file 1 [file Table_1.docx]

**Supplementary Table 1** Comprehensive test for the questionnaire of COVID-19-related work stress validation

| Constructs | items | Reliability | | KMO and  Bartlett  Test of Sphericity | Convergent Validity | | Construct correlation | | | Results of the confirmatory factor analysis |
| --- | --- | --- | --- | --- | --- | --- | --- | --- | --- | --- |
|  |  | Cronbach's Alpha | CR |  | factor loadings | AVE | Effort | Reward | Overcommitment |  |
| Effort | E1 | 0.905 | 0.906 | 0.751  *P* <0.001 | 0.882 | 0.762 | **0.873** |  |  | χ2/df=4.267，*P*＜0.001，GFI=0.942，CFI=0.964，TLI=0.956，NFI=0.954，SRMR=0.068，RMSEA=0.062 |
|  | E2 |  |  |  | 0.841 |  |  |  |  |  |
|  | E3 |  |  |  | 0.895 |  |  |  |  |  |
| Reward | R1 | 0.896 | 0.892 | 0.889  *P* <0.001 | 0.671 | 0.513 | -0.011 | **0.716** |  |  |
|  | R2 |  |  |  | 0.587 |  |  |  |  |  |
|  | R3 |  |  |  | 0.500 |  |  |  |  |  |
|  | R4 |  |  |  | 0.751 |  |  |  |  |  |
|  | R5 |  |  |  | 0.788 |  |  |  |  |  |
|  | R6 |  |  |  | 0.840 |  |  |  |  |  |
|  | R7 |  |  |  | 0.839 |  |  |  |  |  |
|  | R8 |  |  |  | 0.684 |  |  |  |  |  |
| Overcommitment | OC1 | 0.871 | 0.878 | 0.804  *P* <0.001 | 0.680 | 0.646 | 0.671** | 0.063 | **0.804** |  |
|  | OC2 |  |  |  | 0.891 |  |  |  |  |  |
|  | OC3 |  |  |  | 0.890 |  |  |  |  |  |
|  | OC4 |  |  |  | 0.733 |  |  |  |  |  |

Note:***P*＜0.01 CR: Composite Reliability; KMO: Kaiser-Meyer-Olkin; AVE: Average Variance Extracted. In terms of discriminant validity and construct correlation values, the square root of Average Variance Extracted in bold on diagonal, and other values represent correlation coefficient. GFI: Goodness of Fit Index; CFI: Comparative Fit Index; TLI: Tucker-Lewis Index; NFI: Normative Fit Index; SRMR: standardized residual mean root; RMSEA: Root Mean Square Error of Approximation
