# Supplementary material for: The Impact of COVID-19-Related Work Stress on the Mental Health of Primary Healthcare Workers: The Mediating Effects of Social Support and Resilience
Source: Front Psychol. 2022 Jan 21;12:800183. doi: 10.3389/fpsyg.2021.800183 (PMC8814425; doi:10.3389/fpsyg.2021.800183)
Supplement: Supplementary file 2 [file Table_2.docx]

**Supplementary Table 2** Test of normality of study variables based on One-sample Kolmogorov-Smirnov Test

|  | E-R ratio | OC | Social support | OS | SS | UOS | Resilience | PPF | NPF | Anxiety | Depression |
| --- | --- | --- | --- | --- | --- | --- | --- | --- | --- | --- | --- |
| Test Statistic | 0.116 | 0.148 | 0.032 | 0.086 | 0.053 | 0.154 | 0.098 | 0.144 | 0.141 | 0.072 | 0.094 |
| *P* | ＜0.001 | ＜0.001 | 0.045 | ＜0.001 | ＜0.001 | ＜0.001 | ＜0.001 | ＜0.001 | ＜0.001 | ＜0.001 | ＜0.001 |

Note: E-R ratio, Effort-Reward ratio; OC, overcommitment; OS, objective support; SS, subjective support; UOS, use of support; PPF, positive polarity factor; NPF, negative polarity factor
